# The European Cancer Patient Coalition and its central role in connecting stakeholders to advance patient‐centric solutions in the mission on cancer

**DOI:** 10.1002/1878-0261.12448

**Published:** 2019-02-06

**Authors:** Francesco de Lorenzo, Kathi Apostolidis

**Affiliations:** ^1^ European Cancer Patient Coalition Brussels Belgium

**Keywords:** cancer patients, cancer policy, cancer research, innovation, patient organisation, The European Cancer Patient Coalition

## Abstract

There is an urgent need for solutions to the economic and social inequalities in cancer care that still exist in many European countries. Patient preferences, ‘big data’, mobile digital technology and molecular and genomic profiling are among the innovative research topics that connect cancer patients to comprehensive cancer centres, and link translational research to cancer diagnosis, treatment and care. The question is whether Europe can deliver the complex infrastructure needed for universal coverage and equitable access to cancer care. The European Cancer Patient Coalition (ECPC), the leading ‘umbrella’ cancer patient organisation in Europe, has a central role in bringing the unmet needs of patients with cancer to the forefront of cancer policy, care and research. The ECPC is a respected and reliable partner in the oncology community and has effectively collaborated with institutional stakeholders and organisations, as well as with the European Commission, on cancer research projects and in the development of tools to advance health care and cancer policies at the European and national level. The ECPC believes that innovation cannot emerge and grow without patient involvement and is fully committed to increasing patient education and contribution in cancer research through its active participation in various European cancer research programmes and educational resources. The ECPC is expected to play a major role in the mission on cancer, given its previous achievements in policy and research to help overcome the inequalities in cancer prevention, treatment, rehabilitation and survivorship care. The mission on cancer will be facilitated by active collaboration between patient organisations and scientists, clinicians, politicians and industry, with the aim of identifying important research questions regarding quality of life and social issues for cancer patients of all ages.

**Abbreviations**
AGM: Annual General Meeting
ASyMS: Advanced Symptom Management System
BBMRI‐ERIC: Biobanking and BioMolecular resources Research infrastructure
CanCon: Cancer Control
CDDF: Cancer Drug Development Forum
DIAdIC: Dyadic Psychosocial Interventions for people with Advanced cancer and their Informal Caregivers
EACS: European Academy of Cancer Sciences
EAPM: European Alliance for Personalised Medicine
EAU: European Association of Urology
ECCO: European Cancer Conference
ECIBC: European Commission Initiative on Breast Cancer
ECPC: European Cancer Patient Coalition
eHealth: electronic processes supporting healthcare processes
ELBA: European Liquid Biopsy Academy
EMA: European Medicines Agency
ENCePP: European Network of Centres for Pharmacoepidemiology and Pharmacovigilance
ENVI: European Parliament's Committee on Environment, Public Health and Food Safety
EORTC: European Organisation for Research and Treatment of Cancer
EP: European Parliament
EPAAC: European Partnership for Action Against Cancer
ERN: European Reference Network
eSMART: electronic Symptom Management using the Advanced Symptom Management System (ASyMS) Remote Technology
ESMO: European Society for Medical Oncology
ESSO: European Society of Surgical Oncology
EU: European Union
EURACAN: European Network for Rare Adult Solid Cancers
HTA: health technology assessment
IMI: innovative Medicines Initiative
iPAAC: innovative Partnership for Action Against Cancer
JARC: Joint Action on Rare Cancers
MEP: Member of European Parliament
mHealth: mobile digital technologies supporting healthcare processes
OECI: Organisation of European Cancer Institutes
PREFER: Patient Preferences in Benefit‐Risk Assessments during the Drug Life Cycle
PRO: patient‐reported outcome
QOL: quality of life
SABR: stereotactic ablative radiotherapy
UICC: Union for International Cancer Control
WIN: Worldwide Innovative Networking

## 1 Introduction

Europe has played an important role in cancer control. Over the past 30 years, the European Union (EU) has worked alongside its Member States and various stakeholders to control the disease through adequate cancer research and cancer policy. Despite advances in these areas, several unmet needs remain, such as knowledge gaps between patients’ outcomes and clinical practice, analysis of the added value of innovation to patients in the real‐world setting, timely implementation of innovation in clinical cancer pathways and the overall assessment of the quality of cancer care. Patient involvement in research and treatment decisions is a vital contribution that can influence and impact the advancement of cancer management and care.

The European Cancer Patient Coalition (ECPC), established in 2003, is Europe's largest ‘umbrella organisation’, consisting of almost 450 cancer patient organisations in 46 countries. The vision of ECPC is to work towards a Europe of equality, where all European cancer patients have timely and affordable access to the best treatment and care available throughout their life. The main priorities of the ECPC are listed in Box 1.

Box 1 **The main priorities of the European Cancer Patient Coalition (ECPC).**

1

**Table:** 

The ECPC main priorities
Advocacy: cancer policy, work with the European Parliament and European Commission position papers, awareness Capacity building: working groups, education, practical toolkits, annual congress Research: EU projects such as Patient Preferences in Benefit‐Risk Assessments during the Drug Life Cycle (PREFER), electronic Symptom Management using the Advanced Symptom Management System (ASyMS) Remote Technology (eSMART) and Big Data for Better Outcomes Partnerships: representatives in the European Medicines Agency (EMA), the European Network of Centres for Pharmacoepidemiology and Pharmacovigilance (ENCePP), the European Academy of Cancer Sciences (EACS) and Cancer Core Europe's Board of Directors, signed memoranda of understanding with European scientific societies, active partner in many of the European Commission's Joint Actions

The ECPC plays an essential role in Europe by effectively acting as the voice of cancer patients. The organisation is committed to representing patients’ interests and proposing patient‐centric solutions to cancer‐related problems. As such, the ECPC has been a critical stakeholder in defining cancer policy in Europe. Importantly, the ECPC has been raising awareness of the key issues that lead to disparities in cancer care; it has also been making practical recommendations to bridge the gap between cancer policy and cancer care practice. Supported by the ECPC capacity‐building strategy, its member patient organisations have the vital role of advocating for cancer policy recommendations at the national level.

The ECPC has worked with the European Commission on the joint actions against cancer, the European Partnership for Action Against Cancer (EPAAC) and the Joint Action on Cancer Control (CanCon), to standardise the approach of reducing the cancer burden in Europe through the development of guidelines for the harmonisation of National Cancer Plans. The CanCon Guide, a comprehensive, patient‐centric document and a vital tool for governments and policymakers to improve cancer care, is the main deliverable of CanCon that represents an important example of European collaboration in cancer control. This is because each of the cooperating 17 EU Member States and their respective Ministries of Health were directly involved in developing the final recommendations and policies in collaboration with several other stakeholders. The ECPC was a diligent partner in this enterprise, playing a pivotal role in connecting cancer patients with other stakeholders. The ECPC enabled patients to share their own experiences, and their views were included in the very core of the CanCon methodology, work model and final outcomes. As such, the Joint Action on Cancer Control can be viewed as a milestone in cancer policy for all European patients with cancer.

**Figure 1:**
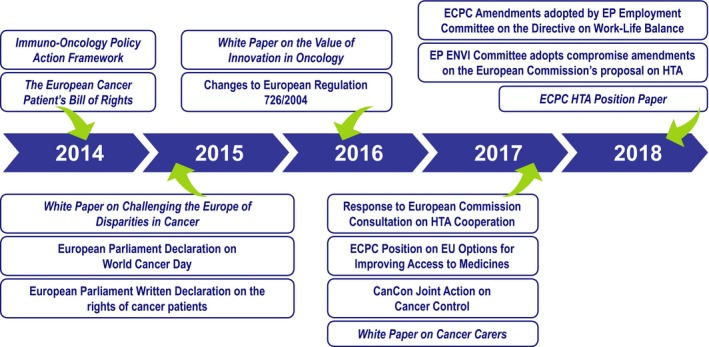
The European Cancer Patient Coalition's (ECPC's) advocacy milestones. ENVI, European Parliament's Committee on Environment, Public Health and Food Safety; EP, European Parliament; HTA, health technology assessment.

At the level of the European Parliament, the European Cancer Patient's Bill of Rights was launched on World Cancer Day 2014 by the European Cancer Concord and the ECPC. This Bill was the result of an equal partnership between patients, their advocates and cancer health professionals. It was considered a ‘catalyst for change’ that recognises the right of each European citizen to optimal and timely access to specialised cancer care, underpinned by research and innovation (Højgaard *et al*., 2017; Lawler *et al*., 2014). The ECPC has also collaborated closely with the European Parliament to promote key cancer policies that have been successfully implemented [such as the development of amendments to health technology assessment (HTA) regulations to harmonise HTA at the EU level, a policy recommendation first outlined in the ECPC position paper ‘Challenging the Europe of disparities in cancer’ in 2015 (European Cancer Patient Coalition, 2015; Fig.**** 1)], highlighting that patients play a crucial role in advancing policies.

The ECPC has worked with a significant number of oncology and cancer care stakeholders to build a broad consensus on issues important to European cancer patients. These partnerships continue to effectively foster a European community of like‐minded cancer experts. The level of cooperation in CanCon and other European Commission cancer‐related initiatives (Table**** 1; Albreht *et al*., 2017; Grilli *et al*., 2017; Peiró Pérez *et al*., 2017) should serve as a model on how to involve and interact with patient organisations. The ECPC has also established multiple key alliances between patients and relevant stakeholders/organisations (Table**** 2; European Cancer Patient Coalition, 2018a). It has further sought to improve the level of collaboration and communication of cancer patients and families with the cancer centres in which they are treated. The active cooperation of patients and families with European cancer centres through open 2‐way communication was the objective of the ECPC's collaboration with the Organisation of European Cancer Institutes (OECI), which resulted in joint initiatives of ECPC members with OECI‐certified comprehensive cancer centres.

**Table 1:** Summary of the European Cancer Patient Coalition's (ECPC's) partnerships with EU institutions

European Commission	Summary of partnership
European Partnership for Action Against Cancer (EPAAC) www.epaac.eu	The first Joint Action was launched in 2009 and ran until 2014. The ECPC played a central role in the survivorship research task.
Joint Action on Cancer Control (CanCon) www.cancercontrol.eu	CanCon ran from 2014 to 2017. The ECPC was a co‐author on the chapter on ‘Survivorship and rehabilitation: policy recommendations for quality improvement in cancer survivorship and rehabilitation in EU Member States’ (Albreht *et al*., 2017), the Policy Paper on ‘Tackling social inequalities in cancer prevention and control for the European population’ (Peiró Pérez *et al*., 2017) and the Policy Paper on ‘Enhancing the value of cancer care through a more appropriate use of healthcare interventions’ (Grilli *et al*., 2017).
Innovative Partnership for Action Against Cancer (iPAAC) https://www.ipaac.eu	The ECPC is playing a central role in this new Joint Action and is actively participating in the Genomics, Cancer Information, and Registries and Challenges in Cancer Care Work Packages.
Joint Action on Rare Cancers (JARC) www.jointactionrarecancers.eu	The ECPC represents the voice of patients with rare cancer within the JARC, with the goal of advancing quality of care and research on rare cancers, specifically European Reference Networks. The ECPC is actively contributing across all Work Packages.
European Reference Networks‐European Network for Rare Adult Solid Cancers (EURACAN) euracan.ern‐net.eu	The ECPC is one of the patient organisations representing the needs, rights and hopes of patients with rare solid cancers, as a European Patient Advisory Group Representative under the EURACAN.
European Commission Initiative on Breast Cancer (ECIBC)	The ECPC is involved in this initiative to update the European Council Recommendation on Cancer Screening.
Expert Group on Cancer Control	The ECPC has representatives in the Expert Group, which was established in 2016 to assist with the preparation of legislative proposals and policy initiatives concerning cancer.

**Table 2:** The European Cancer Patient Coalition's (ECPC's) key partnerships

Scientific societies and organisations	Summary of partnership
European Society for Medical Oncology (ESMO)	Signed ‘Memorandum of Understanding’ in 2016 to enhance cooperation. Strong collaboration formed through updating the ESMO Clinical Practice Guidelines, Patient Guides and the ESMO Handbook on Cancer Survivorship.
Union for International Cancer Control (UICC)	Since 2015, ECPC is a Full Member of UICC and is identifying areas for future collaboration in Europe and globally.
Organisation for European Cancer Institutes (OECI)	Signed ‘Memorandum of Understanding’ in 2015: creation, launch and implementation of a Joint Declaration on Good Relational Practices in Cancer Care and Research. The Joint Declaration outlined the vision that ECPC and OECI share regarding how patients and cancer centres shall interact to enhance patients’ quality of life.
European Organisation for Research and Treatment of Cancer (EORTC)	Signed ‘Memorandum of Understanding’ in 2017. Collaborations ranging from cancer‐related policy issues to patients’ awareness and empowerment. Every 2 years, ECPC and EORTC jointly organise the Cancer Clinical research methodology Course for Patient Advocates.
European Association of Urology (EAU)	Signed ‘Memorandum of Understanding’ in 2018. Collaboration on EAU Patient Information project on Bladder Cancer on the basis of patients’ feedback.
European Alliance for Personalised Medicine (EAPM)	ECPC is a founding member of EAPM and is a key partner in its initiatives. ECPC and EAPM are leading a ‘Personalised Medicine Awareness Month’ campaign.
Cancer Drug Development Forum (CDDF)	A representative of the ECPC sits on the board of the CDDF.
European Society of Surgical Oncology (ESSO)	ESSO contributed to the ECPC document ‘Living well during cancer treatment’ (European Cancer Patient Coalition, 2018a) and ECPC nominated patient advocates to join the ESSO Patients’ Advisory Committee.
Biobanking and BioMolecular resources Research infrastructure (BBMRI‐ERIC)	ECPC is a member of the BBMRI‐ERIC Stakeholder Forum. The 2 organisations are collaborating to translate Italian guidelines for biobanking informed consent to the European level.
Worldwide Innovative Networking (WIN) Consortium	Two representatives from the ECPC sit on the General Assembly of the Worldwide Innovative Networking Consortium.

The ECPC is involved in several European research projects (Table**** 3) and collaborates with the consortium Cancer Core Europe, a patient‐centred infrastructure that aims to address the cancer care‐cancer research continuum challenge in partnership with major European accredited cancer centres (Eggermont *et al*., 2014). The role of the ECPC in Cancer Core Europe is to bridge the gap between cancer research and high‐quality cancer care, strengthened through quality assurance accreditation, and link both to cancer policy. This role is enhanced through the ECPC's involvement in several important EU‐funded cancer research projects. Herein, we further describe the achievements and aims of patient organisations to demonstrate how they can play a major role in the mission on cancer.

**Table 3:** Main EU health and research programmes involving the ECPC, confirmed and ongoing in 2018

EU programme	Objectives
European Reference Networks (ERNs)–European Network for Rare Adult Solid Cancers (EURACAN)	To establish virtual networks connecting healthcare providers and other stakeholders across Europe with the aim to tackle complex or rare diseases and conditions requiring highly specialised treatment and a wealth of knowledge and resources.
European Liquid Biopsy Academy (ELBA)	To educate and provide researchers with skills required to ensure effective development and commercialisation of liquid biopsy approaches.
PREDICT Innovative Training Network	To educate researchers in the fields of radiomics and personalised medicine.
Nutrition in cancer care	To analyse the dimension of nutritional alterations among cancer patients and survivors in Europe (Muscaritoli *et al.*, 2017)
**Funded by Innovative Medicines Initiative (IMI)**	
PREFER patient preferences	To strengthen patient‐centric decision making throughout the life cycle of medicinal products by developing evidence‐based recommendations to guide industry, regulatory authorities, health technology assessment (HTA) bodies, reimbursement agencies, academia and healthcare professionals on how and when patient‐preference studies should be performed, and the results used to support and inform decision making.
DO‐IT Big Data for Better Outcomes	To facilitate the use of ‘big data’ to promote the development of value‐based, outcome‐focused healthcare systems in Europe.
PIONEER Big Data for Better Outcomes	To use big data to improve outcomes for prostate cancer.
**Funded by Horizon 2020**	
Transcan‐2 European Research Area	To contribute to the building of the European Research Area through the coordination of activities of national and regional translational cancer research funding organisations, aiming at the integration of basic, clinical and epidemiologic cancer research and facilitation of transnational cancer funding in Europe with the ultimate aim to streamline EU‐wide cancer screening, early diagnosis, prognosis, treatment and care.
DENIM H2020MM04	To demonstrate the efficacy of dendritic cell‐based immunotherapy in a randomised phase 2–3 clinical trial in mesothelioma.
ImmunoSABR	Randomised, open‐label phase 2 clinical trial; stereotactic ablative radiotherapy (SABR) will be combined with L19‐IL2 immuno‐oncology therapy in patients with limited metastatic non‐small‐cell lung cancer (clinicaltrials.gov no. NCT02735850).
IMMUNISA	In a multicentre, randomised phase 2 clinical trial called CervISA‐2, IMMUNISA will investigate whether a therapeutic cancer vaccine in combination with chemotherapy can prolong the progression‐free survival and overall survival of patients with recurrent or metastatic cervical cancer.
LEGACy	Precision medicine in gastric cancer.
DIAdIC	Dyadic psychosocial interventions.
**Seventh Framework Programme (FP7)**	
eSMART	The clinical trial aims to evaluate the impact of a mobile phone‐based, remote‐monitoring, symptom‐management intervention (the Advanced Symptom Management System, ASyMS) on the delivery of care to people diagnosed with nonmetastatic breast, colorectal or haematologic cancer during chemotherapy and for 1 year after the end of treatment.

## 2 Patients’ inequalities, priorities, and innovative solutions

Patient‐centric approaches in research, medical education, and cancer management and care delivery are critical to an evolving paradigm in cancer care. Currently, various unacceptable inequalities exist across Europe regarding cancer care and rehabilitation and reintegration of people with cancer and cancer survivors in social and professional life. Such inequalities include unequal access to curative cancer treatments; extreme variability and accessibility in cancer screening services; lack of patient‐accessible, accurate, and up‐to‐date information; fragmented or missing cancer rehabilitation services; poor governance; major organisational, structural, and fiscal deficits in health planning; and the lack of a citizen‐focused European cancer survivorship plan [addressed in the ECPC position paper ‘Challenging the Europe of disparities in cancer’ (European Cancer Patient Coalition, 2015)] (Table**** 4; Albreht *et al*., 2017; De Angelis *et al*., 2014; European Cancer Patient Coalition, 2015, 2017a,2017b, 2018a,2018b; European Commission, 2018; European Society for Medical Oncology, 2017; Kandolf Sekulovic *et al*., 2017; Koutoukidis *et al*., 2018; Luengo‐Fernandez *et al*., 2013; Mehnert *et al*., 2013; Peiró Pérez *et al*., 2017).

**Table 4:** Summary of main patient problems and inequalities, and solutions supported by the European Cancer Patient Coalition (ECPC)

Main patient problems/inequalities	Key examples	ECPC recommendations, actions, and solutions
Access to quality care	Patient survival of many cancers in Eastern Europe is below the European average, for example, there is an approximate 60% survival rate for colorectal cancer in Northern and Central Europe, compared with 55% and 45% in Southern and Eastern Europe, respectively (De Angelis *et al*., 2014)	ECPC position paper ‘Challenging the Europe of disparities in cancer’ (European Cancer Patient Coalition, 2015) o Includes a set of policy recommendations on how to address inequalities in terms of multidisciplinary healthcare teams, screening and early diagnosis, cancer registries, access to optimal care, cancer health literacy, cancer survivorship and patient rehabilitation CanCon's Policy Paper ‘Tackling social inequalities in cancer prevention and control for the European population’ (Peiró Pérez *et al*., 2017) o Includes recommendations on addressing social inequalities and disinvestment ECPC value of innovation white paper (‘The value of innovation in oncology’; European Cancer Patient Coalition, 2017a) o Focuses on how to increase access to innovative healthcare solutions for every patient o Includes recommendations on integrating patient‐centred approaches into the regulatory and healthcare system ECPC input on health technology assessment (HTA) and amendments (European Cancer Patient Coalition, 2018b; European Commission, 2018)
Access to medicines	Delays in time to approval of life‐saving treatment, for example, up to 12 years for trastuzumab approval in some countries (Ades *et al*., 2014) Approximately one‐third of patients with metastatic melanoma do not have access to life‐saving innovative treatment (Kandolf Sekulovic *et al*., 2017)	
Sustainability	Healthcare costs of cancer vary substantially in different EU countries, for example, in 2009, healthcare costs ranged from €16 per person in Bulgaria to €184 per person in Luxembourg (Luengo‐Fernandez *et al*., 2013)	
Survivorship and rehabilitation	Survivorship integrates both somatic and psychosocial rehabilitation; therefore, it should encompass physical, psychological and sexual factors, nutritional rehabilitation and practical strategies on topics such as return to work or access to loans, mortgages and health insurance. Cancer patients often face workplace discrimination and even loss of employment, leading to financial and social burdens (Mehnert *et al*., 2013).	CanCon recommendations on survivorship o Includes recommendations on cancer survivors’ health follow‐up plans, late effects management and cancer prevention that need to be tailored to the patient needs and integrated in cancer care with involvement of both survivors and relatives (Albreht *et al*., 2017) The European Society for Medical Oncology (ESMO)‐ECPC guide on survivorship (European Society for Medical Oncology, 2017)
Supportive care	Cancer patients may be insufficiently informed on how additional lifestyle changes, such as exercise, healthy diet and smoking and alcohol cessation, can improve clinical outcomes and overall quality of life (Koutoukidis *et al*., 2018)	The ECPC ‘Living well during cancer treatment’ booklet for people with cancer and their families (European Cancer Patient Coalition, 2018a)
Work–life balance for cancercarers	Informal carers provide 80% of care in Europe, representing a huge cost‐saving for healthcare systems (European Cancer Patient Coalition, 2017b)	The White Paper on Cancer Carers provides a set of policy recommendations for European and National policymakers (European Cancer Patient Coalition, 2017b)

The ECPC has persuaded the participating EU Member States to recognise the need for a Policy Paper on inequalities to complement the CanCon Guide. The ECPC co‐authored the CanCon's Policy Paper ‘Tackling social inequalities in cancer prevention and control for the European population’ (Peiró Pérez *et al*., 2017), which has been signed by the 17 Member States. In brief, the paper advocates cancer diagnosis and treatment in comprehensive centres of excellence to ensure that patients benefit from high‐quality multidisciplinary approaches, surgical expertise, and technological advances, thus increasing their chances of survival. The ECPC also recommends timely access to screening programmes and early diagnosis as preventative strategies.

In line with the CanCon recommendations, EU Member States should establish or update and implement National Cancer Plans, which are currently lacking or are inadequately resourced in many EU countries. Population‐based national cancer registries and biobanking are the basic tools for planning and formulating national cancer and national cancer research policies. A multidisciplinary team‐based strategy for cancer should be adopted by all Member States, providing a patient‐centred approach that addresses all aspects of the cancer journey. Together with the ECPC, all cancer stakeholders should jointly or individually exert pressure on national governments of Member States to ensure implementation of the CanCon policy recommendations.

Access to new treatments remains one of the most significant inequalities across Europe. Currently, patients with cancer face the paradox of life‐saving new treatments becoming available but not accessible to them, depending on which Member State they reside in. There are significant variations in pricing and reimbursement decisions made by EU national governments, which twinned with the lack of a harmonised HTA policy (Ades *et al*., 2014) results in unacceptable delays in access to new treatments. If pricing and/or reimbursement methods remain as they currently are, patient access to innovative treatments such as immunotherapy and biologics and new biomarker testing methodologies and companion diagnostic kits will continue to involve difficulties. This will lead to even greater inequalities than previously experienced.

The ECPC has created momentum on EU cooperation in HTA during the process of amending regulation 726/2004, ‘laying down Community procedures for the authorisation and supervision of medicinal products for human and veterinary use and establishing a European Medicines Agency’, when the proposed amendments were voted by the European Parliament in March 2016. The ECPC response to the European Commission's 2017 consultation on ‘Strengthening of the EU cooperation on Health Technology Assessment’ stated that increased EU cooperation will cut costs and duplication of efforts across HTA bodies, decrease the delay in access to innovative health technologies, facilitate participation of patient organisations in the HTA decision‐making process and facilitate access to market.

## 3 Patient‐centred research

The primary missions of the ECPC are to educate patients, increase their involvement in research and to disseminate information about cancer and cancer research results to the general public. This active engagement of patients is vital to improving the quality of health care in Europe and to understanding patient preferences about their treatment and care, and they are crucial elements of patient‐centred care.

Research projects allow the ECPC to be at the forefront of scientific developments, and the organisation is actively involved in the design and implementation of various EU‐funded projects (summarised in Table**** 3). Within these projects, the ECPC emphasises the importance of involving patients as coresearchers and strongly advocates for patients’ early participation in defining research priorities and patient‐centric treatment decisions. The ECPC encourages a strong partnership between researchers, cancer centres and patients that allows the latter to share their unique experiences, with the aim of achieving a more patient‐centric approach to cancer research and care. The ECPC offers several resources as part of its capacity‐building strategy to guide its member organisations on the crucial role of patients in clinical trials and research programmes. An important project to highlight the advancement of patients’ involvement in research is the Innovative Medicines Initiative (IMI) Project Patient Preferences in Benefit‐Risk Assessments during the Drug Life Cycle (PREFER). The initiative aims to provide evidence‐based recommendations to support guidelines development on how patient preferences should be assessed to inform decision making (de Bekker‐Grob *et al*., 2017).

## 4 Sustainability

To ensure the sustainability and access to innovative cancer care and treatment in European healthcare systems, resources must be secured through budget increases and/or reallocation of resources to interventions and treatments meaningful to patients. To achieve this, current medical and healthcare practices should be continuously re‐evaluated to identify which practices are delivering sufficient value to patients in the most cost‐effective way for health systems.

This approach is outlined in the CanCon Policy Paper ‘Enhancing the value of cancer care through a more appropriate use of healthcare interventions’, which the ECPC co‐authored. The paper includes recommendations on improving access to medicines, surgery, and radiotherapy by reducing waste and improving efficiency (Grilli *et al*., 2017), as well as making use of technology to improve cancer care. The ECPC's contribution helped to define the methodology used to identify healthcare interventions that do not provide sufficient value to patients with cancer and healthcare systems.

## 5 Survivorship and rehabilitation

Cancer survivorship can often be a lifelong struggle. In Europe, more than 3 million people are diagnosed with cancer every year, and nearly 2% of Europeans have been diagnosed with cancer within the previous 6 years (Ferlay *et al*., 2013). Approximately one‐quarter of people who are living after a cancer diagnosis have already reached the same life expectancy as the general population (AIRTUM Working Group, 2014), and the proportion of people who will survive following a diagnosis of cancer is increasing by 3% each year (Guzzinati *et al*., 2018). For example, in Italy, cancer is now the most common noncommunicable disease responsible for disability pensions, with an even higher incidence than cardiovascular disease (Li Ranzi *et al*., 2013).

Despite these increasing numbers, almost all EU countries lack adequate policies to ensure rehabilitation and smooth reintegration of cancer survivors into social and professional life. At least one‐quarter of cancer survivors face long‐term poor health and disability. The increasing number of cancer survivors who require a disability pension impacts the health and social support systems of national governments.

Healthcare providers need to be made more aware of the importance of strategies to improve patient rehabilitation processes, as these are a crucial factor of successful survivorship (Baili *et al*., 2013; De Lorenzo *et al*., 2018). Cancer survivorship often focuses on the distinct phase of cancer care that takes place after the end of the acute phase of cancer treatment and may encompass the physical, mental and/or social aspects of living with, and after, a life‐threatening cancer diagnosis. Consequently, although substantial progress has been made in survivorship research, especially in the area of immediate, persistent and late‐term effects of cancer treatments, more research is needed into the early or late effects of cancer and its treatment, which may cause physical and psychosocial morbidity as well as premature death.

Following the adoption of new treatments in clinical guidelines and current clinical practice, long‐term follow‐up of patients receiving such treatments is required to study patient‐reported outcomes (PROs) and their impact on the quality of life (QOL) of patients. The ECPC has contributed to the recommendations on survivorship, psychosocial support and rehabilitation already developed by the EPAAC and to those generated by CanCon in a chapter in the CanCon guide titled ‘Survivorship and rehabilitation: policy recommendations for quality improvement in cancer survivorship and rehabilitation in EU Member States’ (Albreht *et al*., 2017), which has been endorsed by all 17 participating EU Member States. These recommendations are provided for the development of quality national Survivorship Care Plan (Albreht *et al*., 2017). This guidance offers actionable solutions for the rising number of patients surviving cancer and should serve as a valuable resource for all EU countries. While acknowledging that survivorship care should be an integral part of National Cancer Plans, the recommendations also underscore that more research on survivorship is required to generate data on late effects, as well as on the impact and cost‐effectiveness of supportive care, rehabilitation, and palliative and psychosocial care interventions.

Following the release of the CanCon recommendations, and through its existing collaboration with the European Society for Medical Oncology (ESMO; Table**** 2; European Cancer Patient Coalition, 2018a), the ECPC proposed that a survivorship care follow‐up plan should be put in place for all European patients with cancer. This resulted in the adoption and publication of the Survivorship Guide (ESMO Patient Guide Series. European Society for Medical Oncology, 2017) and was the first time that ESMO recognised the need for such a resource. The purpose of the Survivorship Care Plan, which has its roots in the CanCon Survivorship Work Package and the sustained ECPC advocacy efforts throughout the years, is to offer physicians and patients a concise but comprehensive summary of patient diagnosis and treatment in addition to follow‐up care. The guide bridges the gap between the oncologist and the family doctor and empowers the person with cancer to ask the right questions to their healthcare team. It includes a section on the management of tumour‐related symptoms and rehabilitation, as well as a survivorship checklist, to facilitate continuous care and return to a normal life. The intention is that vital information about a patient's cancer history is readily available to any oncologist or physician the patient may need to consult in the future. The ultimate objective is that the Survivorship Cancer Treatment Summary should become an integral part of the discharge instructions of cancer patients throughout Europe. The Survivorship Guide is a prime example of how the ECPC effectively links cancer policy to clinical practice.

## 6 Supportive care

Nutritional and metabolic disorders are prevalent among cancer patients. As oncologists focus on treating cancer, other important aspects of the disease and effects of cancer treatment are often neglected. Many patients with cancer face challenges related to nutrition, physical activity and health issues that are associated with their treatment and not adequately addressed by their healthcare team.

Cancer‐related nutritional issues, such as malnutrition and cachexia, can substantially impact a patient's QOL, and awareness surrounding these issues is suboptimal. The ECPC has been bringing nutrition in cancer to the attention of patients, health professionals and politicians and is advocating for the right to nutritional support for cancer patients (Caccialanza *et al*., 2017).

Indeed, the ECPC pan‐European nutrition survey aimed to analyse the dimension of nutritional alterations among patients with cancer and survivors by using a structured questionnaire encompassing the perspectives of patients and their physicians on nutritional issues. A total of 92% of patients with cancer did not receive information about cachexia, and 65% stated that their doctors rarely or never checked their weight (Muscaritoli *et al.*, 2017).

Rehabilitation care including plastic surgery, logotherapy, swallowing and breathing training, artificial limbs, and cancer ulcers and ostomy care is not considered part of cancer care in many EU Member States. After the acute phase of treatment, patients are often left on their own to recover basic life skills. Rehabilitation should start immediately upon interventional cancer care (e.g. surgery, radiotherapy, highly toxic systemic treatments) so that the patient can start their recovery without delay. The ECPC believes that integrative cancer care that offers rehabilitation services should be part of the cancer care continuum.

In Europe, long‐term follow‐up data on the effect of treatment on physical and psychosocial functioning or health‐related QOL are still lacking or are fragmented for most cancer diagnoses. These long‐term data are important for providing a comprehensive understanding of the outcome of innovations in diagnostics and treatments, as well as of the quality of cancer care. Knowledge and understanding of these long‐term outcomes are important to clinicians, to healthcare policymakers, to patients who are either cured or have no evidence of active disease and to those living with cancer as a chronic condition. Also, many patients want to know what more they can do on their own to improve their health and well‐being after diagnosis. As a result, more research is required on the factors that help cancer survivors return to normal life, to prevent a secondary tumour and relapses and to prevent the appearance of late effects deriving from cancer treatment, to ensure a smooth process of rehabilitation.

## 7 Work–life balance for cancer carers

The multifaceted burden experienced by carers of patients with cancer has a significant effect on the well‐being of carers, although support for the related financial and psychosocial effects remains inadequate in the EU. Carers often must acquire technical caring skills rapidly with no educational support to learn these skills. In the EU, the cost of unpaid care provided by relatives or friends is approximately €23 billion per year (Luengo‐Fernandez *et al*., 2013). The EU Work‐Life Balance Directive for Parents and Carers proposed by the European Commission clearly outlines the provisions to recognise and protect carers’ right to flexible working conditions and paid caring leave.

The critical role of carers within social and healthcare systems, as well as communities, is recognised by the ECPC and advocates for formal recognition of carers, for implementation of policies that facilitate flexible work arrangements and for training and psychological support to be provided. The ECPC, in collaboration with Members of the European Parliament, proposed amendments to the Work‐Life Balance Directive, which have now been included in the Directive and include formal recognition of the carer. This action highlights a major achievement for the rights of informal caregivers for people with cancer in Europe.

## 8 Digital health

Digital health, which is the combination of digital technologies with health care, aims to improve health and healthcare systems by enhancing accessibility, effectiveness, efficiency and personalisation of care. Mobile technologies are extensively used in hospital systems and should improve supportive care processes. Electronic processes supporting healthcare processes (eHealth) and mobile digital technologies (mHealth) are information and communication technologies that are applied to care pathways to collect patient data (Nasi *et al*., 2015). These technologies can help advance patient‐centric healthcare systems by providing innovative ways for patients to monitor their health and well‐being and by providing patients with increased access to information. This information can be shared with their healthcare providers.

The ECPC is very involved in the mHealth EU‐funded, Seventh Framework Programme (FP7) project electronic Symptom Management using the Advanced Symptom Management System (ASyMS) Remote Technology (eSMART; Table**** 3). This is a clinical trial that aims to evaluate the impact of a mobile phone‐based, remote‐monitoring, symptom‐management intervention (ASyMS) on PROs and the delivery of care. Patients have a prominent coresearcher role in eSMART and have been participating in all project activities from project conception to implementation, including the writing of the trial protocol (Maguire *et al*., 2017).

Digital health is becoming increasingly integrated into the healthcare systems, and it should be recognised that cancer centres participating in Cancer Core Europe have incorporated several aspects of digital health in a wide spectrum of their services. The Internet and digital mobile technologies have brought tremendous changes in how data are transferred, stored, processed and communicated to patients and how patients communicate among themselves and with their physicians. Moreover, the Internet has empowered patients to learn about their disease and ask their healthcare providers questions.

## 9 Molecular testing and personalised medicine

Molecular diagnostics, involving genomic testing and immunotyping, are prerequisites for personalised medicine and are becoming a reality throughout Europe. However, it remains to be seen whether the EU Member States are able to build the complex infrastructure required to support personalised medicine. There is a need for DNA‐sequencing facilities, biobanks for storing samples, ‘big data’ and interoperability solutions, as well as the integration of electronic patient records. Furthermore, health professionals need to be trained to ensure that they can interpret the results of the new tests and data and communicate them effectively to their patients.

The age of personalised medicine is well underway, and further advancement is needed from one‐off assessments to real‐time oncology, where liquid biopsies, artificial intelligence and new companion diagnostics are fully deployed. Multiple targets in each tumour require multiplexed testing, but despite promising results, there are many obstacles facing patients’ access to new biomarker testing and treatments. New companion diagnostics are not available in all EU Member States, and if they are available, they may not be reimbursed, as EU national markets are highly fragmented and lack a consistent approach to HTA both in medicines and companion diagnostics.

Personalised medicine is essential not only for cancer treatment, but also for cancer prevention. Healthcare professionals are increasingly able to identify whether a person is at higher risk for developing cancer via knowledge of mutations commonly linked to cancer and access to more cost‐effective genetic testing.

The concept of molecular and genomic testing is still largely unknown to most patients, and they are often unaware that these newer options are available. Also, healthcare professionals often hesitate to inform patients about new diagnostic and therapeutic options because they are not available or reimbursed, or due to lack of confidence and knowledge regarding genomic and molecular testing. The need for continuing education for healthcare professionals of all oncology specialties is necessary as an increasing number of new biomarker tests and treatments become available. Similarly, information suitable for cancer patients and the general public is needed, to keep pace with the latest scientific and technological developments. The role of the comprehensive cancer centres in Cancer Core Europe and the OECI and of patient organisations in the ECPC will be important in disseminating such information about the new and more complex approaches to cancer diagnosis and treatment (see also other articles in this issue).

The ECPC continuously and diligently aims to offer to its member organisations and the general public information validated by renowned cancer experts about cancer and cancer treatments. To raise patient awareness for molecular testing, the ECPC collaborated with a team of European experts in immuno‐oncology to deliver basic facts in a user‐friendly format to patients, the public and physicians via its immuno‐oncology portal ( http://iop.ecpc.org/; European Cancer Patient Coalition). Moreover, in an effort to make HTA more easily accessible to cancer patients, health professionals and the general public, the ECPC is currently developing an online educational module that aims to equip users with the knowledge to participate in the HTA of cancer treatments. The ECPC has also launched several awareness campaigns to raise the profile of several cancers by sharing facts, statistics, risk factors and symptoms on social media.

## 10 Conclusions and future perspectives

Cancer patients have a growing role in bridging the gap between cancer research, cancer care centres and survivorship and will play an important role moving forward in patients’ and society's ultimate goals: cure and care for cancer patients. Despite successes such as the increasing role of patients in research (e.g. in the IMI‐PREFER and the FP7 eSMART research projects) and the crucial role the ECPC has had in integrating disparities in cancer care, survivorship care and research in the deliverables of the Joint Action CanCon, there is still work to be done before true patient centricity will be achieved in Europe. Central to achieving this goal is ensuring that patients and patient organisations are genuinely involved in the decision‐making processes, which can be achieved by formalising their participation in professional bodies that develop cancer policy recommendations at national and European levels. The key future challenge for the ECPC is to ensure that these recommendations are recognised, adopted and implemented.

A truly patient‐centric healthcare system requires a multidisciplinary approach that is based on scientific guidance readily available to clinicians. With the complexity of cancer biology and the fast‐paced advances in oncology, up‐to‐date guidelines are increasingly important to oncologists to help them improve the quality of their daily practice. As the multidisciplinary approach to cancer diagnosis and care is considered the gold standard, state‐of‐the‐art clinical guidelines describing the essentials of multidisciplinary treatment, and how these can be evidence‐based to ensure adoption of innovative strategies in cancer care, are necessary; these guidelines are currently lacking and it is expected that Cancer Core Europe in collaboration with the other cancer care stakeholders will undertake to develop them. Defining cancer clinical pathways that encompass the continuum of care is even more important and should be developed with input from patients and cancer care providers. European comprehensive cancer centres in collaboration with relevant learned societies, such as the ESMO, the European Society of Surgical Oncology (ESSO) and the European Society for Radiotherapy and Oncology (ESTRO), are the logical key players in coordinating the development and updating of these guidelines. This collaborative effort should facilitate the adoption of multidisciplinary oncology care pathways by the healthcare organisations, thereby improving the standard of care for every patient, encouraging the uptake of innovative research and ensuring the continuum of research from bench to bedside.

Comprehensive cancer centres now offer many of the latest cancer diagnostics and treatments via clinical trials or in the clinical setting. However, access to these centres can be daunting for patients and there is a drive for collaboration between hospitals and comprehensive cancer centres to facilitate the sharing of expertise and resources and increase dissemination of information and best clinical practices. Moreover, partnerships between cancer centres and smaller hospitals should be encouraged to promote knowledge transfer and enable patients to obtain access to specialised services near their home.

It is expected that the Innovative Partnership for Action Against Cancer (iPAAC), the third joint action following on from CanCon that has policy and science at its core, will build upon the deliverables of the Joint Action CanCon and implement innovative approaches to cancer control. A Roadmap on Implementation and Sustainability of Cancer Control Actions will be the main deliverable of this Joint Action and the ECPC will participate in the work packages relating to genomics, cancer information and registries, and challenges in cancer care. In particular, the aim of the genomics work package is to develop practical guidance for EU Member States on important aspects of successful integration of genomics in the healthcare system.

The ECPC's alliances with scientific societies promote a close and patient‐centric collaboration among all stakeholders. The ECPC advocates the use of the CanCon Guide as a roadmap to implement national cancer care plans and to translate policy into practice. The important contribution of the ECPC to cancer research has been recognised by having ECPC representatives appointed to the Scientific Committee of the European Academy of Cancer Sciences (EACS), Cancer Core Europe, board of the Cancer Drug Development Forum (CDDF) and, to the General Assembly of the Worldwide Innovative Networking (WIN) Consortium. Moving forward, it is hoped that the European Commission will continue to sustain and increase its engagement in the fight against cancer and to collaborate with the Member States for the recognition and adoption of the CanCon recommendations.

While cancer research has revolutionised cancer care, it has also created problems, particularly with regards to affordability as many patients in the EU still cannot access innovative life‐saving treatments and diagnostic tests. Currently, the oncology community is somewhat fragmented, a fact that has impacted on its ability to cooperate and effectively counteract the increasing global burden of cancer. The mission‐oriented approach to cancer recently proposed by Celis and Pavalkis (Celis and Pavalkis, 2017), which has been endorsed by members of the oncology community in Europe, has provided a novel strategy to overcome the burden. A primary aim of the mission on cancer is to achieve the long‐term survival of 75% of patients with cancer by 2030 through combining innovative prevention and treatment strategies, as well as rehabilitation and multidisciplinary follow‐up, in a sustainable, contemporary, virtual European cancer infrastructure that has tremendous social and economic implications. The establishment of both Cancer Core Europe (Eggermont *et al*., 2014) and Cancer Prevention Europe (Forman *et al*., 2018) has been key to this endeavour (see also other articles in this issue), and the active participation of the ECPC in the infrastructure should act as a bridge between research and care, strengthening the social impact of translational research and integrating patients’ views into research and cancer care.

Member States and the general public should be involved in the mission on cancer in a coherent and systemic way, encompassing quality assurance and accreditation. The ECPC strongly supports the deployment of quality assurance schemes and the accreditation of cancer centres and to this purpose closely collaborates with the OECI. Dissemination of information to the general public on the genomics revolution in cancer diagnosis and treatment by comprehensive cancer centres and patient organisations will facilitate this process. Patient organisations will collaborate with scientists, clinicians, politicians and industry to overcome the unacceptable disparities in cancer treatment and care, and the associated stigma by identifying important research questions concerning QOL and social issues for all cancer patients, regardless of age. The ECPC can and does facilitate these collaborations and consequently plays a central role in the mission on cancer.

## Author contributions

FDL contributed to the conception and design of the paper, produced the outline, critically reviewed the paper, and approved the final version of the paper. KA contributed to the conception of the paper, critically reviewed the paper and approved the final version of the paper.

## Conflict of interest

The authors declare no conflict of interest.

## References

[mol212448-bib-0001] Ades F , Senterre C , Zardavas D , de Azambuja E , Popescu R , Parent F and Piccart M (2014) An exploratory analysis of the factors leading to delays in cancer drug reimbursement in the European Union: the trastuzumab case. Eur J Cancer 50, 3089–3097. 25446375 10.1016/j.ejca.2014.09.011

[mol212448-bib-0002] AIRTUM Working Group (2014) Italian cancer figures, report 2014: prevalence and cure of cancer in Italy. Epidemiol Prev 38(6 suppl 1), 1–122. 25759295

[mol212448-bib-0003] Albreht T , Andrés JMB , Dalmas M , De Lorenzo F , Ferrari C , Honing C , Huovinen R , Kaasa S , Kiasuwa R , Knudsen AK *et al* (2017) Survivorship and rehabilitation: policy recommendations for quality improvement in cancer survivorship and rehabilitation in EU Member States In European Guide on Quality Improvement in Comprehensive Cancer Control (Albreht T, Kiasuwa R and Van den Bulcke M, eds), pp. 135–164. National Institute of Public Health, Slovenia and Scientific Institute of Public Health, Belgium.

[mol212448-bib-0004] Baili P , Hoekstra‐Weebers J , Van Hoof E , Bartsch HH , Travado L , Garami M , Di Salvo F , Micheli A , Veerus P (2013) Cancer rehabilitation indicators for Europe. Eur J Cancer 49, 1356–1364. 23237740 10.1016/j.ejca.2012.10.028

[mol212448-bib-0005] de Bekker‐Grob EW , Berlin C , Levitan B , Raza K , Christoforidi K , Cleemput I , Pelouchova J , Enzmann H , Cook N , Hansson MG (2017) Giving patients’ preferences a voice in medical treatment life cycle: the PREFER Public‐Private Project. Patient 10, 263–266. 28247251 10.1007/s40271-017-0222-3

[mol212448-bib-0006] Caccialanza R , De Lorenzo F , Gianotti L , Zagonel V , Gavazzi C , Farina G , Cotogni P , Cinieri S , Cereda E , Marchetti P *et al* (2017) Nutritional support for cancer patients: still a neglected right? Support Care Cancer 25, 3001–3004. 28710645 10.1007/s00520-017-3826-1

[mol212448-bib-0007] Celis JE and Pavalkis D (2017) A mission‐oriented approach to cancer in Europe: a joint mission/vision 2030. Mol Oncol 11, 1661–1672. 29024497 10.1002/1878-0261.12143 PMC5709667

[mol212448-bib-0008] De Angelis R , Sant M , Coleman MP , Francisci S , Baili P , Pierannunzio D , Trama A , Visser O , Brenner H and Ardanaz E *et al.* (2014) Cancer survival in Europe 1999–2007 by country and age: results of EUROCARE–5‐a population‐based study. Lancet Oncol 15, 23–34. 24314615 10.1016/S1470-2045(13)70546-1

[mol212448-bib-0009] De Lorenzo F , Apostolidis K , Florindi F and Makaroff LE (2018) Improving European policy to support cancer survivors. J Cancer Policy 15, 72–75.

[mol212448-bib-0010] Eggermont AM , Caldas C , Ringborg U , Medema R , Tabernero J and Wiestler O (2014) Cancer Core Europe: a consortium to address the cancer care‐cancer research continuum challenge. Eur J Cancer 50, 2745–2756. 25263570 10.1016/j.ejca.2014.07.025

[mol212448-bib-0011] European Cancer Patient Coalition (2015) Challenging the Europe of disparities in cancer. http://www.ecpc.org/activities/policy-and-advocacy/policy-initiatives/europe-of-disparities (accessed 7 Nov 2018).

[mol212448-bib-0012] European Cancer Patient Coalition (2017a) The value of innovation in oncology. http://ecpc.org/innovation.pdf (accessed 7 Nov 2018).

[mol212448-bib-0013] European Cancer Patient Coalition (2017b) White paper on cancer carers. http://www.ecpc.org/WhitePaperOnCancerCarers.pdf (accessed 7 Nov 2018).

[mol212448-bib-0014] European Cancer Patient Coalition (2018a) Living well during cancer treatment. http://www.ecpc.org/images/Nutrition_Booklet-Living_well_during_Cancer_Treatment.pdf (accessed 7 Nov 2018).

[mol212448-bib-0015] European Cancer Patient Coalition (2018b) Position on future cooperation on health technology assessment. http://ecpc.org//Documents/ECPC_HTA_Position_Paper_July2018.pdf (accessed 7 Nov 2018).

[mol212448-bib-0016] European Cancer Patient Coalition . ECPC immuno‐oncology portal. http://iop.ecpc.org/ (accessed 7 Nov 2018).

[mol212448-bib-0017] European Commission (2018) Proposal for a regulation of the European parliament and of the Council on health technology assessment and amending directive 2011/24/EU. https://ec.europa.eu/health/sites/health/files/technology_assessment/docs/com2018_51final_en.pdf (accessed 7 Nov 2018).

[mol212448-bib-0018] European Society for Medical Oncology (2017) ESMO Patient Guide Series. Survivorship. https://www.esmo.org/content/download/117593/2061518/file/ESMO-Patient-Guide-Survivorship.pdf (accessed 7 Nov 2018).

[mol212448-bib-0019] Ferlay J , Soerjomataram I , Ervik M , Dikshit R , Eser S , Mathers C , Rebelo M , Parkin DM , Forman D and Bray F (2013) GLOBOCAN 2012: Estimated Cancer Incidence, Mortality and Prevalence Worldwide: IARC CancerBase No. 11. International Agency for Research on Cancer, Lyon, France.

[mol212448-bib-0020] Forman D , Bauld L , Bonanni B , Brenner H , Brown K , Dillner J , Kampman E , Manczuk M , Riboli E , Steindorf K *et al* (2018) Time for a European initiative for research to prevent cancer: a manifesto for Cancer Prevention Europe (CPE). J Cancer Policy 17, 15–23.

[mol212448-bib-0021] Grilli R , Espin J , Florindi F and De Lorenzo F (2017) Policy paper on enhancing the value of cancer care through a more appropriate use of healthcare interventions In Cancer Control Joint Action Policy Papers (Federici A, Nicoletti G and Van den Bulcke M, eds), pp. 29–67. National Institute of Public Health, Slovenia and Scientific Institute of Public Health, Belgium.

[mol212448-bib-0022] Guzzinati S , Virdone S , De Angelis R , Panato C , Buzzoni C , Capocaccia R , Francisci S , Gigli A , Zorzi M , Tagliabue G *et al* (2018) Characteristics of people living in Italy after a cancer diagnosis in 2010 and projections to 2020. BMC Cancer 18, 169. 29426306 10.1186/s12885-018-4053-y PMC5807846

[mol212448-bib-0023] Højgaard L , Löwenberg B , Selby P , Lawler M , Banks I , Law K , Albreht T , Armand JP , Barbacid M , Barzach M *et al* (2017) The European Cancer Patient's Bill of Rights, update and implementation 2016. ESMO Open 1, e000127. 28848664 10.1136/esmoopen-2016-000127 PMC5548978

[mol212448-bib-0024] Kandolf Sekulovic L , Peris K , Hauschild A , Stratigos A , Grob JJ , Nathan P , Dummer R , Forsea AM , Hoeller C , Gogas H *et al* (2017) More than 5000 patients with metastatic melanoma in Europe per year do not have access to recommended first‐line innovative treatments. Eur J Cancer 75, 313–322. 28264791 10.1016/j.ejca.2017.01.012

[mol212448-bib-0025] Koutoukidis DA , Lopes S , Fisher A , Williams K , Croker H and Beeken RJ (2018) Lifestyle advice to cancer survivors: a qualitative study on the perspectives of health professionals. BMJ Open 8, e020313. 10.1136/bmjopen-2017-020313 PMC5875617 29593021

[mol212448-bib-0026] Lawler M , Le Chevalier T , Banks I , Conte P , De Lorenzo F , Meunier F , Pinedo HM , Selby P , Armand JP , Barbacid M *et al* (2014) A Bill of Rights for patients with cancer in Europe. Lancet Oncol 15, 258–260. 24503530 10.1016/S1470-2045(13)70552-7

[mol212448-bib-0027] Li Ranzi T , d'Errico A and Costa G (2013) Association between chronic morbidity and early retirement in Italy. Int Arch Occup Environ Health 86, 295–303. 22476662 10.1007/s00420-012-0765-5

[mol212448-bib-0028] Luengo‐Fernandez R , Leal J , Gray A and Sullivan R (2013) Economic burden of cancer across the European Union: a population‐based cost analysis. Lancet Oncol 14, 1165–1174. 24131614 10.1016/S1470-2045(13)70442-X

[mol212448-bib-0029] Maguire R , Fox PA , McCann L , Miaskowski C , Kotronoulas G , Miller M , Furlong E , Ream E , Armes J , Patiraki E *et al* (2017) The eSMART study protocol: a randomised controlled trial to evaluate electronic symptom management using the advanced symptom management system (ASyMS) remote technology for patients with cancer. BMJ Open 7, e015016. 10.1136/bmjopen-2016-015016 PMC5734219 28592577

[mol212448-bib-0030] Mehnert A , de Boer A and Feuerstein M (2013) Employment challenges for cancer survivors. Cancer 119(suppl 11), 2151–2159. 23695927 10.1002/cncr.28067

[mol212448-bib-0033] Muscaritoli M , Molfino A , Scala F , De Lorenzo F , Christoforidi K and Manneh‐Vangramberen I (2017) European survey of 907 people with cancer about the importance of nutrition. Annals of Oncology 28, v511‐v520.

[mol212448-bib-0031] Nasi G , Cucciniello M and Guerrazzi C (2015) The role of mobile technologies in health care processes: the case of cancer supportive care. J Med Internet Res 17, e26. 25679446 10.2196/jmir.3757 PMC4342745

[mol212448-bib-0032] Peiró Pérez R , Barceló AM , De Lorenzo F , Spadea T , Missinne S , Florindi F , Zengarini N , Apostolidis K , Coleman MP , Allemani C *et al* (2017) Policy paper on tackling social inequalities in cancer prevention and control for the European population In Cancer Control Joint Action Policy Papers (Federici A, Nicoletti G and Van den Bulcke M, eds), pp. 69–110. National Institute of Public Health Slovenia and Scientific Institute of Public Health, Belgium.

